# A case report about oligodendrogliomas of the fourth ventricle

**DOI:** 10.1097/MD.0000000000010594

**Published:** 2018-04-27

**Authors:** Xiujuan Gai, Shaomei Li, Yumei Wei, Shuhua Yu

**Affiliations:** a Department of Oncology, Laiyang Central Hospital of Yantai,Yantai, ShanDong, China; b Shandong Provincial Hospital Affiliated to Shandong University, Shandong Provincial Western Hospital, China.

**Keywords:** fourth ventricle, oligodendrogliomas, prognostic factors, therapy

## Abstract

**Rationale:**

Oligodendrogliomas are usually located in the frontal, parietal and the temporal lobe, with the ones in the fourth ventricle quite rare. Hence we want to introduce a case about the rare disease.

**Patient concerns:**

An eight-year old boy complained of progressive headache, dizziness and vomit for about 2 months. Then the slight ataxia was found by the physical examinations, with no sensory disturbances and other motor disturbances.

**Diagnoses:**

Abnormal signals on the fourth ventricle were found by the preoperative brain computed tomography (CT) scan and magnetic resonance imaging (MRI) scan. So the patient accepted a gross total resection of the lesion with pathologically confirmed oligodendroglioma.

**Interventions:**

Radiotherapy was then delivered in 27 fractions at 2Gy per fraction after the operation, with one fraction daily for five days weekly. No other therapies were used for the patient.

**Outcomes:**

The brain MRI was used for follow-up every three months until now when he has finished all therapies for more than one year. No progressive behaviors (for example, headache, dizziness, vomit and other symptoms about cerebellar tonsillar hernia) or images have been presented. And the follow-up will be continued.

**Lessons:**

Although oligodendrogliomas are usually located in the frontal lobe, with the ones of fourth ventricle extremely rare, they must be kept in mind all times. Treatments applied to our case may be provided as a reference for clinicians. Furthermore, the maximal range of resection, histologically proved oligodendroglioma and the 1p/19q loss of heterozygosity are associated with favorable prognosis.

## 1 Introduction

Oligodendroglioma derives from the oligodendrocyte. It accounts for approximately 1.3% to 4.4% of the brain tumor and about 5% to 10% of the gliocytoma.^[1]^ Only 6% of all diagnosed oligodendrogliomas happened to children and adolescents.^[1]^ They are usually located in or under cerebral cortex, with most of them found in the frontal, parietal and temporal lobe. But primary oligodendroglioma in the ventricle, especially in the fourth ventricle, is quite rare. Herein we would like to introduce a case about the rare disease based on clinical symptoms, treatment, and prognosis.

## 2 Clinical presentation

An 8-year-old boy complained of progressive headache, dizziness and vomit for about 2 months. Then the slight ataxia was found by the physical examinations, with no sensory disturbances and other motor disturbances. And he has no history of seizure or other illnesses.

**Figure 1:**
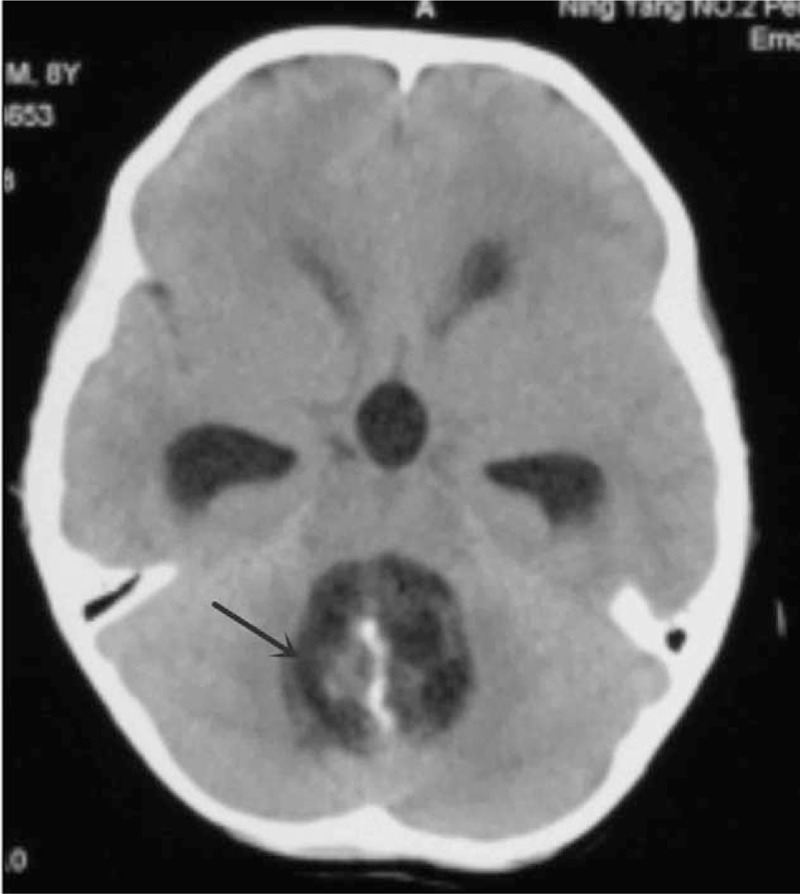
Preoperation CT. CT = computed tomography.

**Figure 2:**
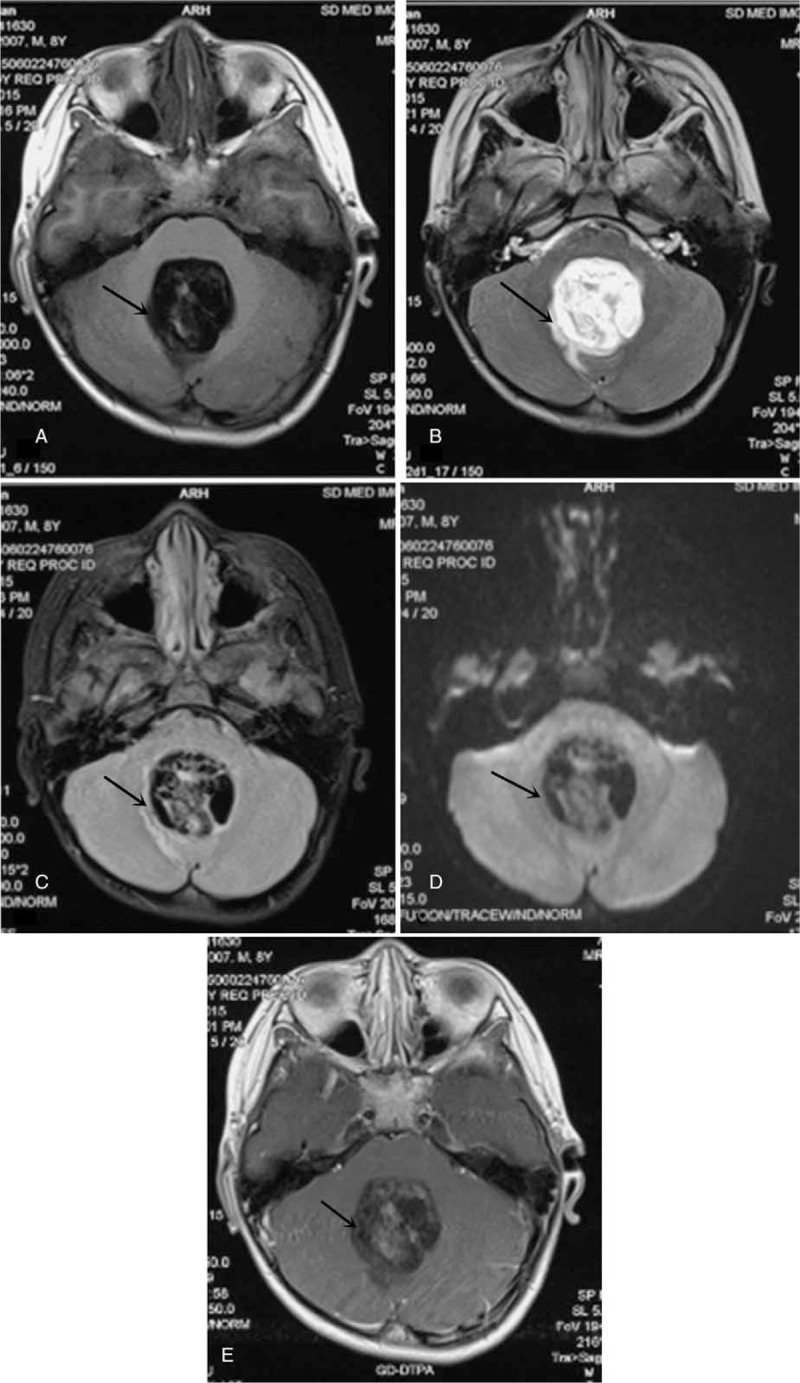
Preoperation MRI (A) T1-weighted sequences. (B) T2-weighted sequences. (C) DWI (*b* = 1000). (D) FLAIR sequences. (E) Image after Gd-DTPA injection. MRI = magnetic resonance imaging, DWI = diffusion weighted imaging, FLAIR = fluid attenuated inversion recovery, Gd-DTPA = gadolinium-diethylenetriamine pentaacetic.

Before surgery, the computed tomography (CT) scan showed that there was a cystic or solid lump with plaque calcification in the fourth ventricle (Fig. 1). The brain magnetic resonance imaging (MRI) showed that in the fourth ventricle there was irregular low signal on T1-weighted sequences (Fig. 2A), high signal on T2-weighted sequences (Fig. 2B), and mixed signal on FLAIR sequences (Fig. 2C). Limited diffusion was not obviously showed on the diffusion weighted imaging (DWI) (*b* = 1000) (Fig. 2D). Mildly heterogeneous reinforcement turned up after the injection of gadolinium-diethylenetriamine pentaacetic (Gd-DTPA) (Fig. 2E). In addition, compression and shift of the brain stem and cerebellum, obvious expansion of lateral ventricles and the third ventricle were also described. No shift of midline structures was seen. All above were concluded as abnormal signals and obstructive hydrocephalus of the fourth ventricle, with the teratogenous tumor or ependymoma more likely.

**Figure 3:**
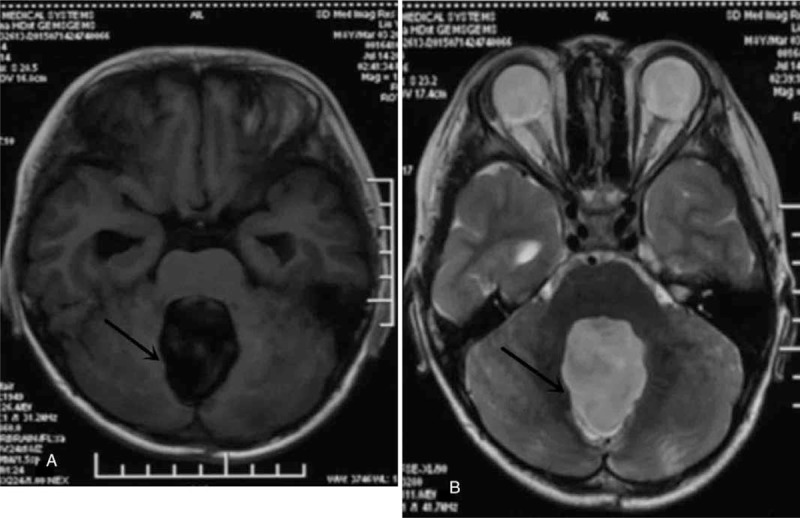
Postoperation MRI A. T1-weighted sequences B. T2-weighted sequences. MRI = magnetic resonance imaging.

Required by his parents, the boy accepted the external ventricular drainage and brain tumor exploratory resection. The range of the operation has reached to the point of gross total resection. Operatively, it was easy to see that the tumor was soft with rich vascular circulation, closely adhering to the floor of the fourth ventricle and extending down to the vermis. The postoperative MRI displayed that structures of surgical area were disordered and partial occipital bone was incontinuous. Moreover, the cerebellar tonsillar was 0.5 cm below the foramen magnum, which signified slight cerebellar tonsillar hernia (Fig. 3).

**Figure 4:**
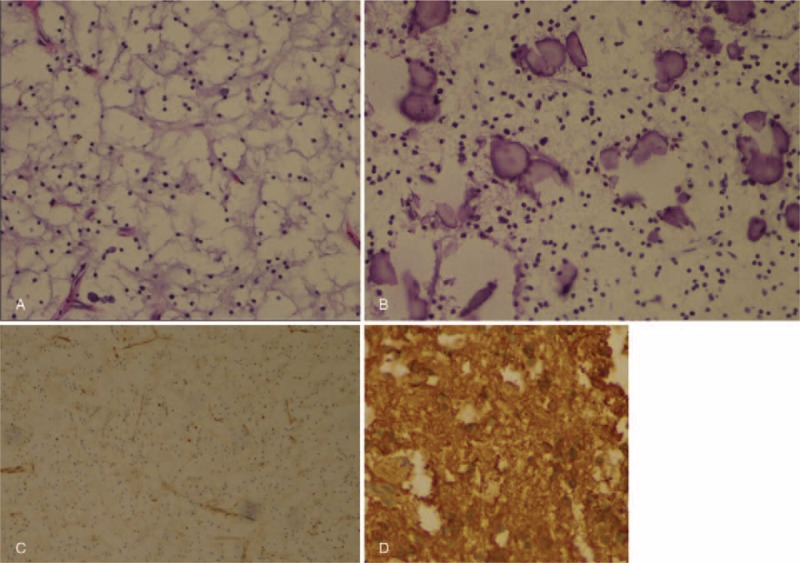
Pathological results of the surgical specimen were observed under a light microscope (Olympus BX51, Tokyo, Japan) and representative pictures were taken. (A) “Fried egg” samples and branching capillaries net represented like chichen toe (HE; original magnification, ×20) (B) Calcification (HE; original magnification, ×20) (C) Olig-2(original magnification, ×20) (D) GFAP (original magnification, ×40). HE = hematoxylin and eosin stain, Olig-2 = oligodendrocyte transcription factor-2, GFAP = glial fibrillary acidic protein.

The histological examination (hematoxylin and eosin stain, HE) of the surgical specimen (Fig. 4A) revealed that tumor cells arranged in medium density. Every cell was comprised of an almost round same size nucleus and bright cytoplasm. The nucleus with obvious karyolemma was located in the center of the cell, which was known as the “fried egg” sample. Branching capillaries net like a chicken toe was also described. In addition, calcification was extensively dispersed (Fig. 4B). Further, immunohistochemical analysis (Fig. 4C and D) showed positive reaction for oligodendrocyte transcription factor-2 (Olig-2) and glial fibrillary acidic protein (GFAP). But as the most commonly biomolecular change, there was no test for the heterozygote of the 1p/19q loss.

In consideration of characteristics of the low-grade gliocytoma and the patient's age, radiotherapy alone was given as the adjuvant therapy after the operation. Intensity modified radiotherapy (IMRT) targeted on the surgical regions was delivered in 27 fractions at 2 Gy per fraction. And the patient was treated with one fraction daily for five days weekly. During the radiation, diplopia and blurred vision occurred. But the symptoms relieved apparently after the utilization of mannitol and dexamethasone. Then brain MRI was used for follow-up every 3 months until now when he has finished all therapies for more than one year. No progressive behaviors (e.g., headache, dizziness, vomit, and other symptoms about cerebellar tonsillar hernia) or images have been presented. And the follow-up will be continued.

The parents of the patient have consented for the publication of the case report.

## 3 Discussion

The overwhelming proportion of primary oligodendrogliomas are located in the cerebral hemisphere, with mostly in the frontal lobes.^[2]^ Only 7% among them are found in the posterior fossa, with one-quarter found in the fourth ventricle,^[3,4]^ to say nothing of the percentage of those happened in children and adolescent. In 1969, Greenwood et al^[3]^ reported 2 cases of adult oligodendrogliomas in the fourth ventricle. Then only another case about a 16-year-old boy was reported by Pitt in 1992.^[5]^

Anatomically, the fourth ventricle lies between the cerebellum and pons, medulla oblongata. It is connected with mesencephalic aqueduct above and cavum subarachnoidale below through the median aperture and 2 lateral apertures of the fourth ventricle. Therefore, tumors growing in the fourth ventricle can affect the flow of cerebrospinal fluid. Further, high intracranial pressure (headache, vomit, and papilloedema) and hydrocephaly are common initial symptoms. They can be attributed to the location. Patients in previous cases showed headache, nausea or blurring of vision, and even blindness.^[3,5]^ Similarly, examinations were performed due to the patient's progressive headache and vomit in our case. Generally, seizure is common with oligodendroglial histology, and it is because the cortex is invaded. No signals of seizure were observed in our case. And obstructive hydrocephalus was verified by the preoperative MRI. Moreover, the postoperative MRI also displayed slight cerebellar tonsillar hernia, mostly for the reason of high intracranial pressure.

Oligodendroglioma of the fourth ventricle belongs to the low-grade (grade II,WHO) gliocytoma (LGG). It is a very benign lesion, particularly when symptoms arise after the age of 21. After Jakola et al^[6]^ reporting their research on the comparison between surgery and biopsy, surgical resection has become the chief choice for LGGs. For those located in nonfunctional areas, the operation principle of maximum range is of consensus. In addition, the extent of resection is correlated significantly with overall survival (OS).^[7,8]^ As tumors growing in the fourth ventricle, a nonfunctional area, partial resection may be relatively easy, but total resection in this region is hard to be realized. It is perhaps followed by secondary changes in adjoining neurological structures, which may be disabled and will not recover.^[3]^ Operation on the boy in our case has reached the principle of maximum resection, a total removal. Although the cerebellar tonsillar hernia after surgery is difficult to relieve, it only need to be observed intently and prevented from progression.

Whether patients with an excellent resection need additional treatment is one of the most contentious areas in neuro-oncology. A prospective study found that more than 50% of patients younger than 40 with gross total resection of LGG recurred within 5 years after operation.^[9]^ So the long-term surveillance imaging is required. Once radiation is elected, a dose of 50 to 54 Gy in 1.8 to 2 Gy daily fraction over 5 to 6 weeks has become standard, with the targets delineated by the tumor area and margin.^[10]^ Significant tumor shrinkage happens to approximately 30% of patients. A random trial (EORTC 22845) has showed that early radiation (54 Gy) could increase the progress-free survival (PFS) rate and decrease the incidence of epilepsy significantly (*P* < .01), but no statistics difference in OS rate was observed.^[11]^ In addition, much of decline in cognitive status has been attributed to radiation therapy. But recent studies have proposed that most cognitive decline in the first few years after radiation is blamed on the tumor characteristic or concomitant medications, such as anticonvulsants.^[12]^ In our case, the lesion is located in a nonfunctional area, less cognitive behavior may appear. Although no reports about it have been showed, the boy was performed with early conventional radiotherapy with a dose of 54 Gy at 2 Gy per once-daily fraction. Apart from diplopia and blurred vision during radiation, which was relieved by mannitol and dexamethasone, no typical decline in cognitive status has been observed till now. Furthermore, the symptom of ataxia was relieved slowly with the radiation going on.

The effect of chemotherapy in patients with LGGs has not been clear until the results of Radiation Therapy Oncology Group (RTOG) 9802 became available. The RTOG 9802 phase III randomized trial demonstrated an increase in PFS rate and a categorical OS benefit (13.3 years vs 7.8 years) for the group of RT armed with PCV compared to those of RT alone.^[13]^ It included newly diagnosed patients with LGGs over age 40 or performed with a subtotal resection. With the emergency of temozolomide (TMZ), a second generation of alkylating agent with low molecular mass, attention has been shifted to this agent. It is a drug of excellent oral bioavailability with plasma concentration up to the peak in one hour and easy to penetrate the blood brain barrier. Whether TMZ can replace radiotherapy and the therapeutic effect combining TMZ with radiotherapy are now research focuses. The RTOG 0424 trial, a single-arm phase II study, has recently reported that for the patients with TMZ and radiotherapy with 3 or more recurrence-related risk factors (age >40 years, histological astrocytoma, both hemispherical tumor, tumor diameter of ≥6 cm preoperative, or the neurological function status preoperative of >1), the 3-year OS rate is higher than that of radiation alone controls.^[14]^ Whether the friendly TMZ can replace PCV remains unknown. A prospective randomized trial may be required to address. Because concurrent chemoradiotherapy has never been reported in patients with total resection, TMZ was not taken orally in our case. Meanwhile, it was not applicable when considering the age of the boy. All examinations demonstrated stable disease till now.

Besides of the range of resection mentioned above, some factors are also connected with prognosis. The RTOG 0424 trial showed that among the risk factors mentioned above only histology was significantly associated with OS (*P* = .0027) and PFS (*P = *.0339).^[14]^ In addition, the prognosis of oligodendroglomas is better than that of the same grade of astrocytomas, and gliomas containing oligodendrocytes are also better in prognosis.^[15]^ Furthermore, the proportion of low-grade oligodendrogliomas turned into high-grade ones (grade III or IV) is about 45% while astrocytomas 74% and oligoastrocytomas 70%, and the median survival time of oliogodendrogliomas is about 8.8 years while astrocytomas 3.1 years and oligoastrocytomas 4.4 years.^[16]^ Meanwhile, the 1p/19q loss of heterozygosity, as the most commonly biomolecular change, is also rewarding for the prognosis of oligodendrogliomas with the incidence about 70%.^[17,18]^ Although there was no test for the heterozygote of the 1p/19q loss in this case, with other favorable factors, presumably the boy will be with a favorable prognosis, and follow-up need be continued.

## 4 Conclusion

Although oligodendrogliomas are usually located in the frontal lobe, with the ones of fourth ventricle extremely rare, they must be kept in mind all times. No reports about them have ever been displayed, but researches on treatments are necessary to focus on. As known from our case, surgery with the maximum range is indispensable. Although additional treatments for patients with an excellent resection have been unresolved, conventional radiotherapy with a dose of 54 Gy at 2 Gy per once-daily fraction without chemotherapy could been considered. Maybe it can be considered as a reference for clinicians. But follow-up and further studies will be required to expand the reach of our observations. In terms of prognosis, the maximal range of resection, histologically proved oligodendroglioma and the 1p/19q loss of heterozygosity are associated with favorable prognosis.

## Author contributions

**Investigation:** Shaomei Li.

**Writing – original draft:** Xiujuan Gai.

**Writing – review & editing:** Yumei Wei, Shuhua Yu.
